# Healthful Plant-Based Diet and Incidence of Type 2 Diabetes in Asian Population

**DOI:** 10.3390/nu14153078

**Published:** 2022-07-27

**Authors:** Jihye Kim, Edward Giovannucci

**Affiliations:** 1 Department of Genetics and Biotechnology, College of Life Sciences, Kyung Hee University, Yongin 17104, Korea; 2 Departments of Epidemiology and Nutrition, Harvard T.H. Chan School of Public Health and Harvard Medical School, Boston, MA 02115, USA; egiovann@hsph.harvard.edu

**Keywords:** plant-based diets, plant food quality, type 2 diabetes, prospective study, Asian

## Abstract

Plant-based diets have been suggested to be beneficial for type 2 diabetes (T2D). However, studies investigating the association between the healthiness of a plant-based diet and T2D risk are limited. This study explored the prospective association between scores from three different plant-based diet indices and risk of T2D and investigated whether associations differ by demographic and lifestyle factors in the Korean population. Data were derived from the Korean Genome and Epidemiology Study (KoGES), a prospective cohort study initiated between 2001 and 2002. Dietary intakes were assessed using a validated food frequency questionnaire. Scores for three plant-based diet indices (overall plant-based diet index (PDI), healthful plant-based diet index (hPDI), and unhealthful plant-based diet index (uPDI)) were measured. A total of 7363 Korean adults aged 40–69 years without T2D and related chronic diseases at baseline were included. Incident T2D was defined as elevated plasma glucose (≥126 mg/dL), self-report of a doctor’s diagnosis of T2D, or use of oral hypoglycemic drug. Multivariable Cox proportional hazards models were used to estimate hazard ratios (HRs) and 95% CIs for T2D risk. During a follow-up period of 14 years, 977 participants developed T2D. A 10-point higher score in hPDI was associated with a 14% lower risk of T2D (HR: 0.86, 95% CI, 0.77–0.95), adjusting for potential confounders. In subgroup analysis, inverse associations between hPDI and T2D risk were stronger in participants with a family history of T2D (HR: 0.58, 95% CI, 0.44 0.76) or history of hypertension (HR: 0.73, 95% CI, 0.60 0.89) than those without a family history of T2D (*p* interaction = 0.01) or history of hypertension (*p* interaction = 0.04). Considering the quality of the plant foods may be important for the prevention of T2D in the Korean population, which habitually consumes diets rich in plant foods.

## 1. Introduction

Type 2 diabetes (T2D) is a major metabolic disorder, which contributes substantially to morbidity and mortality in the world [1]. Diet is a modifiable risk factor in the development of T2D [2]. Plant-based diets have been known to be beneficial for the prevention and management of T2D [3]. Several plant foods, such as fruits, vegetables, whole grains, and legumes, are favorable for the prevention of T2D [4,5,6], but not all plant foods are healthy. For instance, plant foods such as refined grains, sweets, and sugar-sweetened beverages have unfavorable effects on the development of T2D [7,8,9]. Moreover, some animal foods, such as dairy and fish may be beneficial for health outcomes [10,11,12].

The 2015 Dietary Guidelines for Americans recommends gradually moving to diets rich in plant foods and progressively decrease animal food consumption [13]. The plant-based diet indices including overall plant-based diet index (PDI), healthful plant-based diet index (hPDI), and unhealthful plant-based diet index (uPDI), assess intakes of both plant foods and animal foods, taking the quality of plant foods into account [14,15]. In these indices, animal foods are negatively weighed but differ with respect to how plant foods are weighed. Existing studies reported that the scores from PDI or hPDI were associated with a lower risk of cardiovascular disease, T2D, and related chronic diseases [14,15,16,17]. On the contrary, the score from uPDI was positively associated with the risk of these metabolic diseases [14,15,18,19]. However, the study on the association of newly established plant-based diet indices with T2D risk is limited in the Asian population although their eating patterns and metabolic responses may be different from the Western population [20,21]. Asians consume higher amounts of grains and vegetables and lower amounts of meat than Western populations [21]. Different types of grains may affect glucose metabolism differently [22,23]. Previous studies reported ethnic differences in T2D risk associated with scores of a priori-defined dietary patterns [24,25]. One prospective study found that the scores of PDI and hPDI was associated with a reduced risk of type 2 diabetes in Singapore Chinese [16].

Thus, this study evaluated the associations between scores from three different plant-based diet indices (PDI, hPDI, and uPDI) and risk of T2D and investigated whether associations differed by demographic and lifestyle factors in a large community-based cohort of Korean middle-aged and older adults.

## 2. Materials and Methods

### 2.1. Study Population

We used the data from the Korean Genome and Epidemiology Study (KoGES), a population-based cohort study, which aimed to explore the genetic and lifestyle factors of T2D and hypertension among Koreans [26]. A total of 10,030 community residents (40–69 years of age) living in Ansan and Ansung city, near Seoul, were enrolled. The KoGES was initiated between 2001 and 2002 (baseline) and participants were followed up biennially until 2016. The follow-up rate in the KoGES was over 90%. The Institutional Review Boards of the Korea Disease Control and Prevention Agency and Kyung Hee University (KHGIRB-19-398) approved the study protocol, and participants provided written informed consent.

**Figure 1:**
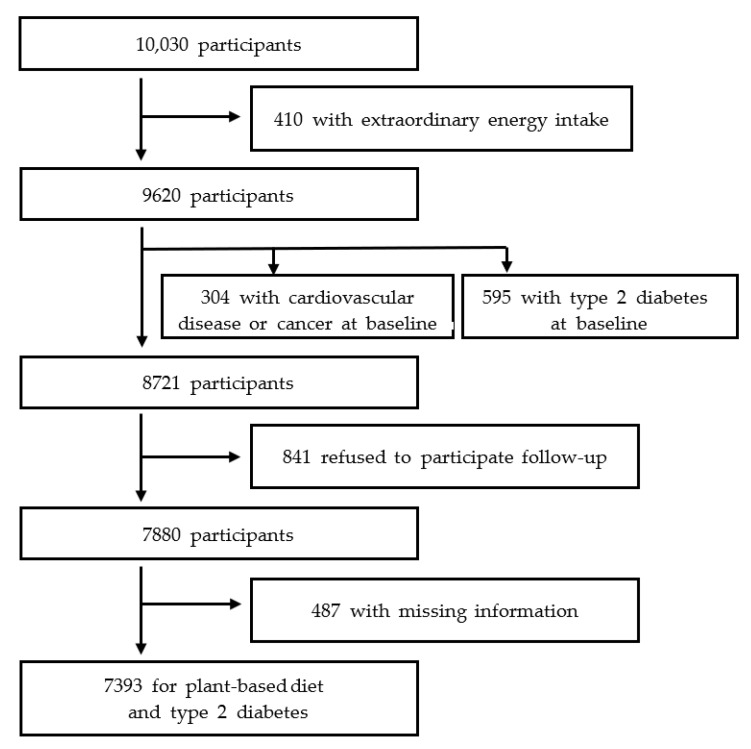
Flow diagram of participant selection.

In the analysis, exclusion criteria included individuals with implausible energy intake at baseline (<500 kcal/d or >5000 kcal/d) (*n* = 410), had cardiovascular disease or cancer at baseline (*n* = 304), did not visit in follow-up examinations (*n* = 841), who had T2D at baseline (*n* = 595), and who had missing data on the outcome of T2D or covariates including education level, physical activity, cigarette smoking, alcohol consumption, baseline body mass index (BMI), total energy intake, family history of T2D, and history of hypertension (*n* = 487). The final analysis included 7393 (3466 men and 3927 women) (Figure 1).

### 2.2. Assessment of Plant-Based Diet Index Score

Participants were asked for their usual food intake with a 106-item semi-quantitative food frequency questionnaire (FFQ). Validity and reproducibility for FFQ have been previously evaluated [27]. The correlation coefficient of nutrient density between the two FFQs examined at a 1-year interval was between 0.22 (vitamin A) and 0.51 (calcium) (average: 0.39). The median value of correlation coefficients for nutrients between the FFQ and the 12-day diet records was 0.39. The FFQ was assessed at baseline and visit 3, which is the second follow-up (visit 3: 2005–2006). We applied the average of dietary intake from two FFQs for the calculation of plant-based diet indices. When participants developed T2D before visit 3 or did not complete the questionnaire at visit 3, we used only baseline dietary intake. Participants were asked to report the frequency and the portion size of food consumption over the past year. The FFQ had nine answers for frequency of consumption, ranging from “almost never” to “3 times or more per day,” and three answers for portion size (small, medium, or large) [28].

We applied previously reported processes for calculating three plant-based diet index scores [15,18] (Table S1). Briefly, the food items were classified into 17 food groups based on nutrient and culinary similarities and then the food groups were categorized into three larger groups, which are healthy, less healthy plant and animal food group. We distinguished between healthy plant foods and less healthy plant foods depending on the associations of food items with disease risk [14,15,18,19]. Healthy plant foods include whole grains, fruits, vegetables, nuts, legumes, tea/coffee, and less healthy plant foods include refined grains, potatoes, sugar-sweetened beverages, sweets and desserts, salty foods. Animal foods include animal fat, dairy, eggs, fish, meat, and miscellaneous animal foods. We classified salty foods (i.e., kimchi) as less healthy plant foods due to high sodium content. We did not separate vegetable oil and fruit juices included in the original indices as food groups [14,15], because the oil intake was not queried in the FFQ, and fruits and fruit juices were queried together. Alcoholic beverages were excluded from the calculation of indices due to unclear directions of association for various health outcomes. Some mixed dishes, such as pizza and hamburgers/sandwiches, were queried individually in the FFQ and were categorized into miscellaneous animal foods.

After we formulated group foods, participants were ranked into energy-adjusted quintiles. For the PDI score, participants in the highest quintile of each of all plant foods were scored 5 while those in the lowest quintile of each of all plant foods were scored 1. For the hPDI, participants in the highest quintile of only healthy plant foods were scored 5 while those in the highest quintile of less healthy plant foods were scored 1. For the uPDI, participants in the highest quintile of only less healthy plant foods were scored 5 while those in the highest quintile of healthy plant foods were scored 1. For all plant-based diet indices, animal foods were adversely scored. For instance, participants in the highest quintile of animal fat consumption were scored 1, and those in the lowest quintile of animal fat consumption were scored 5. Thus, higher scores in all plant-based diet indices represented lower consumption of animal foods. After summing up the scores across these categories for plant and animal foods, the overall diet scores were divided into quintiles for analysis. In the present study, the Spearman correlation coefficients between PDI and hPDI were the highest (0.44), and the correlations were −0.15 between hPDI and uPDI, and 0.14 between PDI and uPDI.

### 2.3. Ascertainment of Type 2 Diabetes

T2D incidence was defined as having one or more of the following criteria [29]: elevated fasting plasma glucose, use of the oral hypoglycemic drug, or current treatment with insulin. Biochemical assessment, medical history, and medication use were identified at biennial follow-up visits. Elevated plasma glucose was considered as ≥126 mg/dL. Blood samples were collected after ≥8 h of fasting and the samples were stored at −80 °C until analyses. An auto-analyzer (ADVIA 1650, Bayer HealthCare) was used to measure the glucose concentration enzymatically using a standardized protocol. In a reliability study, the laboratory value of this biomarker is highly reproducible (Pearson’s correlation > 0.99) [30].

### 2.4. Assessment of Covariates

Information on demographic and lifestyle factors at baseline were investigated using structured questionnaires, administered by trained interviewers. Educational level was divided into ≤6, 7 to 12, and >12 years. Cigarette smoking was queried as pack-years of cigarettes. Alcohol intake was assessed among former and current drinkers who had consumed alcohol within one year. Physical activity was calculated using the metabolic equivalent of task based on the types and intensity of physical activity [31]. Baseline height and weight of participants were measured by trained staff. Height was measured to the nearest 0.1 cm without shoes using a stadiometer (Samhwa Instrument, Seoul, Korea) and body weight was measured to 0.1 kg in light clothes without shoes. Body mass index (BMI) was calculated from measured weight (kg) divided by height squared (m^2^). We calculated total energy intake using a food composition table from the Korean Nutrition Society [32]. History of hypertension at baseline was defined as a self-report of a doctor’s diagnosis of hypertension.

### 2.5. Statistical Analysis

Baseline characteristics of participants are described as mean and standard deviation (SD) or number and percentage (categorical variables). Food group consumption was compared between the lowest and highest quintile of each plant-based diet indices and was expressed as serving size per 1000 kcal.

The risk of T2D per a 10-point higher score of plant-based diet indices were tested using multivariable Cox proportional hazards models. Age (year, continuous) and sex (men/women) were adjusted in Model 1. Additionally, residential location (rural/urban), education (≤6, 7–12, >12 years), physical activity (MET/day, continuous), cigarette smoking (continuous), alcohol consumption (g/day, continuous), BMI (kg/m^2^, continuous), total energy intake (kcal/day, continuous), family history of diabetes (yes/no), and history of hypertension at baseline (yes/no) were adjusted in Model 2. We selected the potential confounding factors from the previous literature [14,18].

Next, the restricted cubic splines with 4 knots were applied to examine the shape of the associations for plant-based diet indices. Effect modification by sex, baseline BMI, family history of diabetes, and history of hypertension were tested with cross-product terms.

Person year was calculated as the time from baseline examination until the date of T2D event or censoring. Censoring was defined as those who did not develop T2D until the end date of the study or those who developed cancer or cardiovascular disease before developing diabetes during follow–up visits. We did not consider the death of participants, due to the unavailability of the data.

We tested the proportional hazard assumption using Schoenfeld’s residuals, which was met [33]. All data were analyzed using SAS software, version 9.4 (SAS Institute, Cary, NC, USA) [34]. *p* < 0.05 was considered significant for two-sided tests.

## 3. Results

During a follow-up of 82,351 person-years, 977 (13%) T2D cases were identified. Table 1 summarizes participants’ characteristics at baseline. Individuals in the Q5 (highest quintile) of PDI and hPDI were more likely to be older, women, to live in rural areas, had lower education levels and cigarette smoking (modest for PDI), consuming less alcohol, had higher BMI, were more physically active, and were more likely to have hypertension compared to those in the Q1 (lowest quintile) of PDI and hPDI. Individuals in the Q5 of uPDI were more likely to be older, men, to live in rural areas, had lower education levels, higher cigarette smoking, had lower BMI, were more physically active, and were more likely to have hypertension compared to those in the Q1 of uPDI.

Table 2 shows the food group consumption of participants in the lowest (Q1) versus highest quintile (Q5) of plant-based diet indices. Individuals in the Q5 of PDI consumed greater amounts of whole grains, fruits, vegetables, legumes, tea and coffee, potatoes, sweets and desserts, and salty foods and less amounts of refined grains, dairy, eggs, fish, and meat compared to those in the Q1 of PDI. Individuals in the Q5 of hPDI consumed greater amounts of whole grains, fruits, vegetables, and legumes and consumed less amounts of refined grains, potatoes, sugar-sweetened beverages, sweets and desserts, and all animal foods compared to those in the Q1 of hPDI. On the contrary, individuals in the Q5 of uPDI consumed less amounts of all healthy plant foods and greater amounts of refined grains and salty foods compared to those in the Q1 of uPDI.

**Figure 2:**
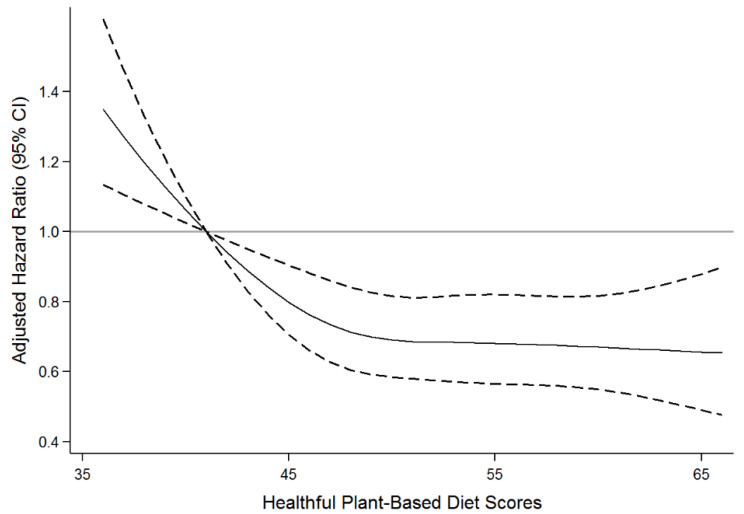
Association of healthful plant-based diet score with incident type 2 diabetes according to the continuous plant-based diet score among the middle-aged and older Korean population.

Table 3 reports the hazard ratios and 95% confidence intervals for developing T2D according to a 10-point higher score of plant-based diet indices. In the age and sex-adjusted model, the hPDI was not significantly associated with the risk of T2D (HR = 0.92, 95% CI, 0.83–1.02). However, in the multivariable-adjusted model, a 10-point higher score of hPDI was associated with a 14 % lower risk of T2D after adjustment for potential confounders (HR: 0.86, 95% CI, 0.77–0.95). When we additionally adjusted for dietary fiber or calcium, the results remained the same. This association was reflected when the relation between hPDI with incident T2D was visually depicted (Figure 2). However, neither PDI nor uPDI was significantly associated with the risk of T2D.

The solid lines represent the multivariable-adjusted hazard ratios for incident type 2 diabetes, modelled using restricted cubic splines with 4 knots (5th, 35th, 65th, and 95th percentiles). The reference was set at the 5th percentile of the score. The dashed lines represent 95% confidence intervals. The model adjusted for age (year, continuous) and sex (men/women), residence area (rural/urban), education (≤6, 7–12, >12 years), physical activity (MET/day, continuous), cigarette smoking (continuous), alcohol intake (g/day, continuous), baseline body mass index (kg/m^2^, continuous), total energy intake (kcal/day, continuous), family history of diabetes (yes/no), and history of hypertension at baseline (yes/no).

Additionally, when we conducted the subgroup analysis stratified by sex, baseline BMI, family history of T2D, and history of hypertension, an inverse relationships between hPDI and T2D risk were stronger in participants with a family history of T2D (HR: 0.58, 95% CI, 0.44 0.76) or history of hypertension (HR: 0.73, 95% CI, 0.60 0.89) than those without a family history of T2D (*p* interaction = 0.01) or history of hypertension (*p* interaction = 0.04). (Table 4)

## 4. Discussion

Greater adherence to a healthful plant-based diet (captured by hPDI) was associated with a 14% lower risk of T2D in South Korean adults after adjustment for demographic factors, lifestyle factors, and BMI. In subgroup analyses, the inverse relationship was stronger among participants with a family history of T2D or history of hypertension than those without a family history of T2D or history of hypertension. However, overall plant-based diets (captured by PDI) and unhealthy plant-based diets (captured by uPDI) were not significantly associated with the risk of T2D. The inverse association of hPDI with the risk of T2D highlights that greater adherence to diets higher in healthy plant foods and lower in less healthy plant foods and animal foods may be important for the prevention of T2D.

Several studies have explored the association between plant-based diets and T2D risk. Three prospective cohort studies have reported that higher score of PDI and hPDI were associated with 49% and 45% lower risk of T2D, respectively, whereas higher score of uPDI was associated with a 16% higher risk of T2D in the US adults [14]. The current study also showed a stronger association of the hPDI with T2D. A Singapore Chinese study showed that higher scores of PDI and hPDI were associated with 17% and 19% lower risk of T2D, respectively, in the middle-aged and older adults [16]. In the Rotterdam study, a higher adherence to an overall plant-based diet was associated with a 13% lower T2D risk after adjustment for potential confounders in Dutch adults [3]. Kim et al. showed that great adherence to PDI was associated with a 20% lower risk of elevated fasting glucose (≥100 mg/dL) as a component of metabolic syndrome, but not with hPDI or uPDI among Korean adults [18]. The differences in findings between the two Korean studies may be due to the differences in exclusion criteria of participants, diagnosis criteria (e.g., blood glucose cut point), and covariates for adjustment, although the same cohort data were used.

Unlike US adults [14], an overall plant-based diet captured by PDI was not associated with the incidence of T2D in the Korean population. No association of PDI with T2D shows that increasing the amount of plant foods without consideration of the quality of plant foods may not be beneficial for the prevention of T2D in a population that habitually consumes diets high in plant foods and low in animal foods. Based on our data, Korean adults consumed half the amount of animal foods and more plant foods than US adults [14]. In addition, the differences in non-dietary lifestyle risk factors for T2D may be attributable to differential effect size in T2D risk between US adults and Korean adults. Korean adults have healthier indicators on average including lower BMI, more physical activity, and less alcohol consumption than US adults [14].

In the current study, participants in the highest quintile of hPDI consumed greater amounts of whole grains, fruits, vegetables, and legumes, and lesser amounts of refined grains, sugar sweetened beverages, animal fat, and meat compared to those in the lowest quintile of hPDI. Combinations of these food groups would contribute to reducing the risk of T2D. In a meta-analysis, whole grains, fruits, vegetables, and soy products were associated with a reduced risk of T2D, while refined grains, red meat, processed meat, and sugar sweetened beverages were associated with an increased risk of T2D [35,36]. Several mechanisms have been suggested for how a healthful plant-based diet lowers the risk of T2D. Diets rich in healthy plant foods include various favorable food components and nutrients such as dietary fiber, antioxidants, micronutrients, and unsaturated fatty acids [37,38], and the diet is low in saturated fatty acids and cholesterol. A meta-analysis of clinical trials has shown that soluble fiber intake improved glycemic control in patients with T2D [39]. Epidemiologic studies have shown flavonoids/polyphenols may have anti-diabetic effects by increasing glucose metabolism, improving vascular functions as well as reducing insulin resistance [40]. Polyunsaturated fatty acids are associated with improved glycemic control and with anti-inflammatory effects [41]. In addition, another mechanism may be related to the gut microbiome. A healthy plant-based diet may improve gut microbiota profiles that facilitate the metabolism of dietary components, such as fiber and polyphenols, and diminish the metabolism of microbial metabolites, such as trimethylamine N-oxide, which are primarily found in animal originated foods [42,43]. This microbial change may induce a reduction of T2D risk.

Interestingly, an inverse association between the healthier version of the plant-based diet and T2D was stronger in participants who have a family history of T2D or history of hypertension. This population may be at a higher risk of T2D, possibly due to abnormal obesity-related body composition (higher BMI and waist-hip ratio) and genetic factors [44]. This suggests that a healthier dietary pattern may have more benefits for a vulnerable population who are at a higher risk of T2D.

This study explored the prospective associations between different plant-based diet indices assessed by the healthiness of plant-based diet and the risk of T2D in the Korean population, which has traditionally consumed diets rich in plant foods. Strengths of this study include the use of data from a community-based cohort, validated FFQ, repeated dietary assessments, and a relatively long follow-up period. The current study also focused on the Korean population which may have different eating habits from the population in Western countries.

However, several limitations should be noted. Reporting of dietary intakes can be subject to measurement error although the FFQ was validated in South Korean adults [45]. The FFQ has some mixed dishes that combine healthy plant food and less healthy plant food and combines plant food and animal food such as Bibimbap. We queried fruit (healthy) and fruit juice (less healthy) together in the FFQ and data on vegetable oil intake was not collected from the FFQ. Furthermore, the processing/cooking method was not considered in the differentiation of healthy plant and less healthy plant foods. These food classifications might not completely distinguish the consumption of healthy plant or less healthy plant foods and, thus, they may have led to an attenuation of the association between uPDI and incident T2D. Although we used the average of dietary intakes from two FFQs, there is possibility that dietary habits of participants might have changed over time. T2D cases might be missing because HbA1c was not included as the criteria of diagnosis. Some person’s time may be misclassified because data on death or moving were not available. Missing data on covariates were not imputed in the current analysis. Lastly, there may still be residual confounding although important confounders were adjusted.

## 5. Conclusions

In a community-based cohort of Korean men and women, greater adherence to diets high in healthy plant foods and low in unhealthy plant foods and animal foods in the context of a plant-based diet was associated with a lower risk of incident T2D. These results emphasize the importance of considering the quality of plant foods for the prevention of T2D in Koreans. Further research confirming the associations between plant-based diet indices and T2D in diverse ethnic populations with different dietary habits are warranted.

## Figures and Tables

**Table 1:** Baseline characteristics of study participants in the lowest versus highest quintile of plant-based diet indices.

	PDI		hPDI		uPDI	
	Quintile 1	Quintile 5	Quintile 1	Quintile 5	Quintile 1	Quintile 5
Sample size, *n*	1451	1438	1506	1452	1586	1186
Median score (range)	44 (31–46)	58 (56–69)	43 (28–45)	59 (57–74)	43 (29–46)	62 (60–76)
Female, *n* (%)	673 (46.4)	875 (60.9)	542 (36.0)	1033 (71.1)	1014 (63.9)	581 (49.0)
Age, years	49.7 (8.4)	53.8 (8.9)	49.2 (8.2)	54.2 (8.7)	49.1 (7.8)	55.4 (8.9)
Residential location, *n* (%)						
Rural, Ansung	618 (42.6)	830 (57.7)	496 (32.9)	834 (57.4)	394 (24.8)	939 (79.2)
Urban, Ansan	833 (57.4)	608 (42.3)	1010 (67.1)	618 (42.6)	1192 (75.2)	247 (20.8)
Education level, *n* (%)						
≤6 years	334 (23.0)	607 (42.2)	315 (20.9)	616 (42.4)	233 (14.7)	627 (52.9)
7–12 years	842 (58.0)	709 (49.3)	886 (58.8)	716 (49.3)	1009 (63.6)	499 (42.1)
>12 years	275 (19.0)	122 (8.5)	305 (20.3)	120 (8.3)	344 (21.7)	60 (5.0)
Cigarette smoking (pack-year)	9.5 (14.9)	8.4 (15.6) *	12.6 (16.4)	5.7 (13.1)	6.3 (13.1)	11.0 (17.1)
Alcohol intake (g/day)	11.8 (23.1)	7.1 (20.1)	13.7 (26.3)	5.7 (17.8)	8.7 (20.7)	8.4 (19.0)
Body Mass Index (kg/m^2^)	24.4 (3.0)	24.8 (3.2)	24.5 (3.1)	24.8 (3.3)	24.7 (3.0)	24.3 (3.2)
Physical activity (MET/day)	21.7 (14.3)	25.9 (15.6)	21.7 (14.0)	24.8 (15.5)	20.2 (12.0)	27.2 (16.8)
Family history of diabetes, *n* (%)	179 (12.3)	140 (9.7) *	160 (10.6)	143 (9.9) *	223 (14.1)	91 (7.7)
History of hypertension, *n* (%)	151 (10.4)	227 (15.8)	160 (10.6)	282 (19.4)	200 (12.6)	203 (17.1)

Data are expressed as mean ± SD or n (%). PDI, overall plant-based diet index; hPDI, healthful plant-based diet index, MET, Metabolic equivalent of task; uPDI, unhealthful plant-based diet index. * Values are not significantly different between Q1 and Q5 group. Except for that, all values are significantly different between Q1 and Q5 group.

**Table 2:** Dietary consumption of participants in the lowest versus highest quintile of plant-based diet indices.

	PDI		hPDI		uPDI	
	Quintile 1	Quintile 5	Quintile 1	Quintile 5	Quintile 1	Quintile 5
Total energy intake, kcal/day	2048 (676)	1836 (584)	2016 (594)	1850 (617)	2064 (584)	1717 (584)
Food group intake (servings/1000 kcal/week)						
Whole grain	4.6 (4.4)	7.0 (4.5)	2.5 (3.2)	9.9 (3.7)	7.5 (3.5)	3.6 (4.5)
Fruits	8.2 (7.0)	13.2 (8.3)	7.4 (5.3)	13.7 (9.2)	14.0 (7.7)	7.0 (6.7)
Vegetables	11.9 (6.7)	18.2 (8.4)	13.5 (6.4)	16.1 (9.3)	18.5 (8.0)	10.6 (6.4)
Nuts	0.1 (0.3)	0.3 (0.5)	0.1 (0.4)	0.3 (0.6)	0.4 (0.7)	0.0 (0.2)
Legumes	1.7 (1.5)	3.8 (3.0)	1.9 (1.6)	3.7 (3.0)	3.3 (2.0)	1.7 (2.2)
Tea and coffee	4.3 (3.8)	6.5 (4.9)	6.2 (4.7)	4.6 (4.4)	6.4 (4.4)	3.9 (4.8)
Refined grains	3.3 (4.6)	1.8 (2.9)	4.7 (5.0)	0.9 (1.6)	1.0 (1.7)	4.6 (5.4)
Potatoes	0.6 (0.6)	1.3 (1.2)	1.0 (0.9)	0.8 (1.0)	0.9 (0.9)	0.9 (1.5) *
Sugar-sweetened beverages	0.6 (1.0)	0.7 (1.0)	1.1 (1.1)	0.3 (0.6)	0.6 (0.8)	0.6 (1.0) *
Sweets and desserts	3.4 (3.0)	4.8 (3.8)	5.6 (3.5)	2.6 (2.9)	3.9 (2.9)	3.8 (4.2) *
Salty foods	11.6 (6.2)	19.6 (8.2)	16.0 (7.5)	15.2 (8.3)	13.3 (6.2)	18.8 (9.3)
Animal fat	3.5 (3.8)	3.5 (4.4) *	5.3 (4.3)	1.9 (3.2)	4.1 (3.9)	2.8 (4.3)
Dairy	3.4 (2.7)	1.8 (2.1)	3.0 (2.3)	2.1 (2.5)	3.8 (2.6)	1.3 (1.9)
Eggs	1.1 (1.0)	0.6 (0.8)	1.1 (0.9)	0.6 (0.8)	1.2 (0.9)	0.4 (0.7)
Fish	4.7 (2.7)	3.7 (2.9)	5.0 (2.7)	3.5 (3.1)	5.9 (2.9)	2.0 (1.7)
Meat	0.9 (1.1)	0.4 (0.5)	1.2 (1.3)	0.3 (0.5)	0.6 (0.7)	0.5 (0.8)
Miscellaneous animal foods	0.2 (0.3)	0.1 (0.2)	0.2 (0.3)	0.1 (0.1)	0.2 (0.2)	0.1 (0.2)
Healthy plant foods	30.8 (12.0)	48.9 (13.6)	31.7 (10.8)	48.3 (14.7)	50.2 (12.5)	26.8 (10.7)
Less healthy plant foods	19.5 (8.4)	28.2 (9.7)	28.3 (9.9)	19.7 (8.8)	19.7 (6.9)	28.8 (11.9)
Animal foods	13.8 (5.7)	10.1 (5.7)	15.8 (5.5)	8.5 (5.3)	15.8 (5.0)	7.0 (4.9)

Values are means (SD). * Values are not significantly different between Q1 and Q5 group. Except for that, all values are significantly different between Q1 and Q5 group.

**Table 3:** Hazard ratios and 95% confidence intervals for incident type 2 diabetes according to a 10-point higher score of plant-based diet indices.

Model	PDI	hPDI	uPDI
Median (SD)	51 (5.2)	51 (6.4)	52 (7.0)
Model 1	1.04 (0.92–1.18)	0.92 (0.83–1.02)	1.06 (0.97–1.16)
Model 2	0.99 (0.88–1.12)	0.86 (0.77–0.95)	1.06 (0.96–1.18)

Model 1 was adjusted for age (year, continuous) and sex (men/women). Model 2 was additionally adjusted for residential area (rural/urban), education (≤6, 7–12, >12 years), physical activity (MET-hour/day, continuous), smoking cigarettes (continuous), alcohol intake (g/day, continuous), baseline body mass index (kg/m^2^, continuous), total energy intake (kcal/day, continuous), family history of diabetes (yes/no), and history of hypertension at baseline (yes/no).

**Table 4:** Hazard ratios (HR) and 95% confidence intervals (CI) for incident type 2 diabetes according to the continuous hPDI score, stratified by selected characteristics.

	Healthful Plant-Based Diet Index	*p* Interaction
Sex		
Men	0.89 (0.77–1.04) *	0.85
Women	0.84 (0.72–0.97)	
Baseline body mass index		
≥25 kg/m^2^	0.84 (0.73–0.96)	0.19
<25 kg/m^2^	0.90 (0.76–1.07)	
Family history of T2D		
Yes	0.58 (0.44–0.76)	0.01
No	0.92 (0.82–1.03)	
History of hypertension		
Yes	0.73 (0.60–0.89)	0.04
No	0.92 (0.81–1.04)	

* per a 10-point higher score of healthful plant-based diet index. Adjusted for age (years, continuous), residence area (rural/urban), education (≤6, 7–12, >12 years), physical activity (MET/day, continuous), cigarette smoking (continuous), alcohol intake (g/day, continuous), body mass index (kg/m^2^, continuous), total energy intake (kcal/day, continuous), family history of diabetes (yes/no), and history of hypertension at baseline (yes/no). MET, metabolic equivalent task; T2D, type 2 diabetes.

## Data Availability

Data underlying the results of our study are not publicly available due to KoGES data policy. Data are available from the Division of Genetic Epidemiology and Health Index, NIH, Korea Disease Control and Prevention Agency (contact via Mi-Jin Cho at whalwls0227@korea.kr) for researchers who meet the criteria for access to confidential data.

## Supplementary Materials

The following are available online at https://www.mdpi.com/article/10.3390/nu14153078/s1, Table S1: Classification of food items in the Korean Genome and Epidemiology Study (KoGES).

## References

[1] Kos K. (2020). Cardiometabolic morbidity and mortality with smoking cessation, review of recommendations for people with diabetes and obesity. Curr. Diab. Rep..

[2] Ley S.H., Hamdy O., Mohan V., Hu F.B. (2014). Prevention and management of type 2 diabetes: Dietary components and nutritional strategies. Lancet.

[3] Chen Z., Zuurmond M.G., van der Schaft N., Nano J., Wijnhoven H.A.H., Ikram M.A., Franco O.H., Voortman T. (2018). Plant versus animal based diets and insulin resistance, prediabetes and type 2 diabetes: The Rotterdam Study. Eur. J. Epidemiol..

[4] Aune D., Norat T., Romundstad P., Vatten L.J. (2013). Whole grain and refined grain consumption and the risk of type 2 diabetes: A systematic review and dose–response meta-analysis of cohort studies. Eur. J. Epidemiol..

[5] Cooper A.J., Forouhi N.G., Ye Z., Buijsse B., Arriola L., Balkau B., Barricarte A., Beulens J.W., Boeing H., Büchner F.L. (2012). Fruit and vegetable intake and type 2 diabetes: EPIC-InterAct prospective study and meta-analysis. Eur. J. Clin. Nutr..

[6] Muraki I., Imamura F., Manson J.E., Hu F.B., Willett W.C., van Dam R.M., Sun Q. (2013). Fruit consumption and risk of type 2 diabetes: Results from three prospective longitudinal cohort studies. BMJ.

[7] Halton T.L., Willett W.C., Liu S., Manson J.E., Stampfer M.J., Hu F.B. (2006). Potato and french fry consumption and risk of type 2 diabetes in women. Am. J. Clin. Nutr..

[8] Malik V.S., Popkin B.M., Bray G.A., Després J.-P., Willett W.C., Hu F.B. (2010). Sugar-sweetened beverages and risk of metabolic syndrome and type 2 diabetes: A meta-analysis. Diabetes Care.

[9] Soriguer F., Colomo N., Olveira G., García-Fuentes E., Esteva I., de Adana M.S.R., Morcillo S., Porras N., Valdés S., Rojo-Martínez G. (2013). White rice consumption and risk of type 2 diabetes. Clin. Nutr..

[10] Gijsbers L., Ding E.L., Malik V.S., De Goede J., Geleijnse J.M., Soedamah-Muthu S.S. (2016). Consumption of dairy foods and diabetes incidence: A dose-response meta-analysis of observational studies. Am. J. Clin. Nutr..

[11] Mozaffarian D. (2016). Dietary and policy priorities for cardiovascular disease, diabetes, and obesity: A comprehensive review. Circulation.

[12] Zhang M., Picard-Deland E., Marette A. (2013). Fish and marine omega-3 polyunsatured fatty acid consumption and incidence of type 2 diabetes: A systematic review and meta-analysis. Int. J. Endocrinol..

[13] US Department of Agriculture, US Department of Health and Human Services (2015). Scientific Report of the 2015 Dietary Guidelines Advisory Committee: Advisory Report to the Secretary of Health and Human Services and the Secretary of Agriculture. Washington (District of Columbia). https://health.gov/our-work/nutrition-physical-activity/dietary-guidelines/previous-dietary-guidelines/2015/advisory-report.

[14] Satija A., Bhupathiraju S.N., Rimm E.B., Spiegelman D., Chiuve S.E., Borgi L., Willett W.C., Manson J.E., Sun Q., Hu F.B. (2016). Plant-based dietary patterns and incidence of type 2 diabetes in US men and women: Results from three prospective cohort studies. PLoS Med..

[15] Satija A., Bhupathiraju S.N., Spiegelman D., Chiuve S.E., Manson J.E., Willett W., Rexrode K.M., Rimm E.B., Hu F.B. (2017). Healthful and unhealthful plant-based diets and the risk of coronary heart disease in US adults. J. Am. Coll. Cardiol..

[16] Chen G.-C., Koh W.-P., Neelakantan N., Yuan J.-M., Qin L.-Q., van Dam R.M. (2018). Diet quality indices and risk of type 2 diabetes mellitus: The Singapore Chinese Health Study. Am. J. Epidemiol..

[17] Kim J., Kim H., Giovannucci E.L. (2021). Quality of plant-based diets and risk of hypertension: A Korean genome and examination study. Eur. J. Nutr..

[18] Kim H., Lee K., Rebholz C.M., Kim J. (2020). Plant-based diets and incident metabolic syndrome: Results from a South Korean prospective cohort study. PLoS Med..

[19] Lee K., Kim H., Rebholz C.M., Kim J. (2021). Association between different types of plant-based diets and risk of dyslipidemia: A prospective cohort study. Nutrients.

[20] Afshin A., Micha R., Khatibzadeh S., Fahimi S., Shi P., Powles J., Singh G., Yakoob M.Y., Abdollahi M., Al-Hooti S. (2015). Global Burden of Diseases I, Risk Factors Study N, Chronic Diseases Expert G, Metabolic Risk Factors of Chronic Diseases Collaborating Group. The impact of dietary habits and metabolic risk factors on cardiovascular and diabetes mortality in countries of the Middle East and North Africa in 2010: A comparative risk assessment analysis. BMJ Open.

[21] Micha R., Khatibzadeh S., Shi P., Andrews K.G., Engell R.E., Mozaffarian D. (2015). Global Burden of Diseases N, Chronic Diseases Expert G. Global, regional and national consumption of major food groups in 1990 and 2010: A systematic analysis including 266 country-specific nutrition surveys worldwide. BMJ Open.

[22] Hopping B.N., Erber E., Grandinetti A., Verheus M., Kolonel L.N., Maskarinec G. (2010). Dietary fiber, magnesium, and glycemic load alter risk of type 2 diabetes in a multiethnic cohort in Hawaii. J. Nutr..

[23] Kolonel L.N., Henderson B.E., Hankin J.H., Nomura A.M., Wilkens L.R., Pike M.C., Stram D.O., Monroe K.R., Earle M.E., Nagamine F.S. (2000). A multiethnic cohort in Hawaii and Los Angeles: Baseline characteristics. Am. J. Epidemiol..

[24] Jacobs S., Harmon B.E., Boushey C.J., Morimoto Y., Wilkens L.R., Le Marchand L., Kröger J., Schulze M.B., Kolonel L.N., Maskarinec G. (2015). A priori-defined diet quality indexes and risk of type 2 diabetes: The Multiethnic Cohort. Diabetologia.

[25] Liese A.D., Nichols M., Sun X., D’Agostino R.B., Haffner S.M. (2009). Adherence to the DASH diet is inversely associated with incidence of type 2 diabetes: The insulin resistance atherosclerosis study. Diabetes Care.

[26] Kim Y., Han B.-G. (2017). KoGES Group. Cohort profile: The Korean genome and epidemiology study (KoGES) consortium. Int. J. Epidemiol..

[27] Ahn Y., Kwon E., Shim J., Park M., Joo Y., Kimm K., Park C., Kim D. (2007). Validation and reproducibility of food frequency questionnaire for Korean genome epidemiologic study. Eur. J. Clin. Nutr..

[28] Korea Disease Control and Prevention Agency (KDCA) (2011). Manual of Korean Genome and Epidemiology Study. Osong: KDCA, National Institute of Health, Center for Genomics. https://nih.go.kr/contents.es?mid=a50401010300#1.

[29] American Diabetes Association (2010). Standards of medical care in diabetes—2010. Diabetes Care.

[30] Yang J.J., Yang J.H., Kim J., Cho L.Y., Park B., Ma S.H., Song S.H., Min W.-K., Kim S.S., Park M.S. (2011). Reliability of quadruplicated serological parameters in the Korean genome and epidemiology study. Epidemiol. Health.

[31] Ainsworth B.E., Haskell W.L., Whitt M.C., Irwin M.L., Swartz A.M., Strath S.J., OBrien W.L., Bassett D.R., Schmitz K.H., Emplaincourt P.O. (2000). Compendium of physical activities: An update of activity codes and MET intensities. Med. Sci. Sports Exerc..

[32] The Korean Nutrition Society (2000). Food Composition Table. Recommended Dietary Allowances for Koreans, 7th ed.

[33] Schoenfeld D. (1982). Partial residuals for the proportional hazards regression model. Biometrika.

[34] (2020). SAS [Computer Program].

[35] Li W., Ruan W., Peng Y., Wang D. (2018). Soy and the risk of type 2 diabetes mellitus: A systematic review and meta-analysis of observational studies. Diabetes Res. Clin. Pract..

[36] Schwingshackl L., Hoffmann G., Lampousi A.M., Knuppel S., Iqbal K., Schwedhelm C., Bechthold A., Schlesinger S., Boeing H. (2017). Food groups and risk of type 2 diabetes mellitus: A systematic review and meta-analysis of prospective studies. Eur. J. Epidemiol..

[37] Schwingshackl L., Schwedhelm C., Hoffmann G., Lampousi A.-M., Knüppel S., Iqbal K., Bechthold A., Schlesinger S., Boeing H. (2017). Food groups and risk of all-cause mortality: A systematic review and meta-analysis of prospective studies. Am. J. Clin. Nutr..

[38] Ma Y., Hébert J.R., Li W., Bertone-Johnson E.R., Olendzki B., Pagoto S.L., Tinker L., Rosal M.C., Ockene I.S., Ockene J.K. (2008). Association between dietary fiber and markers of systemic inflammation in the Women’s Health Initiative Observational Study. Nutrition.

[39] Xie Y., Gou L., Peng M., Zheng J., Chen L. (2020). Effects of soluble fiber supplementation on glycemic control in adults with type 2 diabetes mellitus: A systematic review and meta-analysis of randomized controlled trials. Clin. Nutr..

[40] Cao H., Ou J., Chen L., Zhang Y., Szkudelski T., Delmas D., Daglia M., Xiao J. (2019). Dietary polyphenols and type 2 diabetes: Human study and clinical trial. Crit. Rev. Food Sci. Nutr..

[41] Poreba M., Rostoff P., Siniarski A., Mostowik M., Golebiowska-Wiatrak R., Nessler J., Undas A., Gajos G. (2018). Relationship between polyunsaturated fatty acid composition in serum phospholipids, systemic low-grade inflammation, and glycemic control in patients with type 2 diabetes and atherosclerotic cardiovascular disease. Cardiovasc. Diabetol..

[42] Rinninella E., Cintoni M., Raoul P., Lopetuso L.R., Scaldaferri F., Pulcini G., Miggiano G.A.D., Gasbarrini A., Mele M.C. (2019). Food components and dietary habits: Keys for a healthy gut microbiota composition. Nutrients.

[43] Tomova A., Bukovsky I., Rembert E., Yonas W., Alwarith J., Barnard N.D., Kahleova H. (2019). The effects of vegetarian and vegan diets on gut microbiota. Front. Nutr..

[44] Choi J., Choi J.-Y., Lee S.-A., Lee K.-M., Shin A., Oh J., Park J., Song M., Yang J.J., Lee J.-k. (2019). Association between family history of diabetes and clusters of adherence to healthy behaviors: Cross-sectional results from the Health Examinees-Gem (HEXA-G) study. BMJ Open.

[45] Watanabe D., Yoshida T., Yoshimura E., Nanri H., Goto C., Ishikawa-Takata K., Ebine N., Fujita H., Kimura M., Yamada Y. (2021). Doubly labelled water–calibration approach attenuates the underestimation of energy intake calculated from self-reported dietary assessment data in Japanese older adults. Public Health Nutr..

