# Supplementary material for: Healthful Plant-Based Diet and Incidence of Type 2 Diabetes in Asian Population
Source: Nutrients. 2022 Jul 27;14(15):3078. doi: 10.3390/nu14153078 (PMC9332860; doi:10.3390/nu14153078)
Supplement: Supplementary file 1 [file nutrients-14-03078-s001.zip › nutrients-1819340-supplementary.pdf]

Table S1. Classification of food items in the Korean Genome and Epidemiology Study (KoGES)[1]

Food groups	Items in the food frequency questionnaire
**Healthy plant foods**	
Whole grains	Mixed grains, barley, grain with beans
Fruits	Strawberry, watermelon, banana, peach/ plum, oriental melon/melon, persimmon/dried persimmon, pear/pear juice, tangerine, orange/orange juice, apple/apple juice, grape/grape juice
Vegetables	Sweet potatoes, radish, napa cabbage/napa cabbage soup, spinach, lettuce, perilla leaves, sesame leaves/vegetable salad, other green vegetable, Deodeok/bellflower root, bean sprouts/mung-bean sprouts, bracken/sweet potato stem, oyster mushroom, other mushrooms, green pepper leaf/chamnamul, crown daisy /chive /watercress, cucumber, carrot/carrot juice, onion, green peppers, zucchini, pumpkin/kabocha squash, laver, kelp/seaweed, tomato/tomato juice
Nuts	Peanuts/almonds/pine nuts
Legumes	Beans/beans cooked in soy sauce, tofu, bean curd, soybean milk
Tea and coffee	Coffee, green tea
**Less healthy plant foods**	
Refined grains	White rice, instant noodles, other noodles (udon noodles), black bean sauce noodles, cold noodles, rice cake/rice cake soup, other rice cakes, refined cereals, white breads, other breads, grain powder, starch jelly, stir-fried noodles and vegetables
Potatoes	Potatoes (steamed, french fries, soup, fried potato pancake)
Sugar sweetened beverages	soft drink, other beverages (sweetened rice tea, citron tea)
Sweets and desserts	Sweet red bean bread, cake/chocolate pie, cookies/crackers/snacks, candies/chocolates, sugars (added to tea or coffee)
Salty food group	Bean paste, Bean paste/bean paste soup, Kimchi (Korean cabbage, radish), watery radish kimchi, other kimchi, pickled vegetable (preserved in soy sauce or salt), radish kimchi (preserved in soy sauce or salt)
**Animal foods**	
Animal fat	Butter, cream (added to tea or coffee)
Dairy	Milk, yogurt/yoplait, ice cream, cheese
Eggs	Eggs/quail eggs
Fish	Sashimi, belt fish, mackrele/pacific saury, eel, yellow croaker/sea bream/sole, alaska pollack/frozen pollack/dried pollack, squid/dried squid/octopus, anchovy/stir-fried anchovy, canned tuna, salted shrimp/salted fish, clam/sea snail, oyster, crab/marinated crab, shrimp, fishcake
Meat	Pork belly, grilled pork/stir-fried pork/pork bulgogi/Korean meatball, steamed pork, processed meat (ham,sausage), organ meat/Korean sausage, steak/grilled beef, dog meat, beef soup, chicken(fried, stew, braised spicy chicken), beef stew
Miscellaneous animal foods	dumplings/dumpling soup, pizza/hamburger

[1] The PDI, hPDI, and uPDI categorized food groups to “healthy plant foods,” “less healthy plant foods,” and “animal foods.”

PDI, overall plant-based diet index; hPDI, healthful plant-based diet index; uPDI, unhealthful plant-based diet index
